# Effects of optimism on creativity under approach and avoidance motivation

**DOI:** 10.3389/fnhum.2014.00105

**Published:** 2014-02-28

**Authors:** Tamar Icekson, Marieke Roskes, Simone Moran

**Affiliations:** Guilford Glazer Faculty for Business and Management, Ben Gurion University of the NegevBeer Sheva, Israel

**Keywords:** optimism, motivation, approach, avoidance, creativity

## Abstract

Focusing on avoiding failure or negative outcomes (avoidance motivation) can undermine creativity, due to cognitive (e.g., threat appraisals), affective (e.g., anxiety), and volitional processes (e.g., low intrinsic motivation). This can be problematic for people who are avoidance motivated by nature and in situations in which threats or potential losses are salient. Here, we review the relation between avoidance motivation and creativity, and the processes underlying this relation. We highlight the role of optimism as a potential remedy for the creativity undermining effects of avoidance motivation, due to its impact on the underlying processes. Optimism, expecting to succeed in achieving success or avoiding failure, may reduce negative effects of avoidance motivation, as it eases threat appraisals, anxiety, and disengagement—barriers playing a key role in undermining creativity. People experience these barriers more under avoidance than under approach motivation, and beneficial effects of optimism should therefore be more pronounced under avoidance than approach motivation. Moreover, due to their eagerness, approach motivated people may even be more prone to unrealistic over-optimism and its negative consequences.

In today’s competitive and dynamic world, designing an environment that is optimal for creativity is a main concern of many organizations, workplaces, and educational settings. Creativity—i.e., generating ideas, insights, or solutions that are both novel and useful (Amabile, 1996), is a key ingredient of innovation, and is needed to adapt to changing technologies and demands, and to distinguish oneself or one’s company from others (Oldham and Cummings, 1996; Simonton, 1999). Not surprisingly therefore, scientists and practitioners strive to identify conditions that influence creativity. One critical factor that impacts peoples’ creative performance is the type of goals that drive their behavior. Previous research demonstrates that striving for positive outcomes or success (approach motivation) enhances creativity, whereas striving to avoid negative outcomes or failure (avoidance motivation) undermines it (Friedman and Förster, 2005; Elliot et al., 2009; Mehta and Zhu, 2009).

## Avoidance motivation and creativity

Goals give direction to people’s behavior toward positive outcomes or away from negative outcomes. The goals people adopt are influenced by individual differences; some people tend to focus more on avoidance goals and others more on approach goals (Elliot et al., 1997; Elliot and Thrash, 2010), but goals are also influenced by fluctuating situations. Whereas safe situations in which potential rewards or other positive outcomes are salient typically evoke approach motivation, threatening situations in which potential losses or other negative outcomes are dominant usually evoke avoidance motivation. Compared to approach motivation, avoidance motivation is associated with a host of psychological processes that undermine creativity. Indeed, evidence that avoidance motivation reduces creativity is abundant (e.g., Friedman and Förster, 2002, 2005; Elliot et al., 2009; Mehta and Zhu, 2009; Lichtenfeld et al., 2012). For example, in one study Friedman and Förster (2002) asked people to perform motor actions associated with approach motivation (i.e., arm flexion, a movement resembling bringing objects closer) or avoidance motivation (i.e., arm extension, resembling distancing objects). People in the approach compared to avoidance condition came up with more creative ways for using a brick.

**Figure 1:**
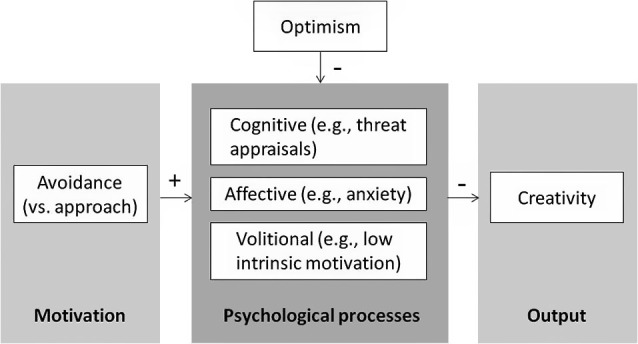
**By influencing the psychological processes that are evoked by avoidance motivation, optimism reduces the undermining effect of avoidance motivation on creativity**.

Because creative solutions can be useful, and sometimes even necessary, to avert threats—for example, to repel an enemy or avoid bankruptcy—the relation between avoidance motivation and decreased creativity can be problematic. Consequently, identifying strategies to reduce the negative effects of avoidance motivation on creativity is important. Addressing the core psychological processes that underlie creativity, we propose that optimism may play a crucial role in remedying these negative effects. Our main argument is that optimism—i.e., expecting to succeed in achieving success or avoiding failure, moderates the relationship between motivational orientation and creativity. Specifically, we expect that optimism reduces the negative effects of avoidance motivation on creativity, as it reduces problematic psychological processes such as threat appraisals, anxiety, and disengagement. Because avoidance motivation is less problematic for non-creative tasks (e.g., those that require attention to detail), the positive effects of optimism should be less pronounced outside the domain of creativity. Additionally, the positive effects of optimism on creativity should be less pronounced when people are approach motivated. Approach motivation may even make people prone to over-optimism, and lead to reduced creativity (see Figure 1 for the theoretical model).

In the following sections, we discuss optimism, approach and avoidance motivation, and creativity, and provide the rationale underlying our model. Specifically, we discuss: (1) the nature and consequences of optimism; (2) the psychological processes associated with approach and avoidance motivation and their relation to creativity; and (3) how optimism taps into each of these processes and moderates the relationship between motivation and creativity.

## What is optimism?

The tendency to positively perceive the future seems to be an inherent aspect of human nature (Varki, 2009; Sharot, 2011). Indeed, people often overestimate potential positive events in their future, while underestimating negative events (e.g., Hoorens et al., 2008; Waters et al., 2011; Shepperd et al., 2013). Scheier and Carver (1985) describe optimism as a generalized tendency to expect positive outcomes even in the face of obstacles. According to this view, optimists expect good things to happen in the future and therefore actively strive to achieve their goals. Buchanan and Seligman (1995) describe optimism in terms of how people explain bad events in their past. According to this view, optimists explain bad events with external, unstable, and specific causes, whereas pessimists explain bad events with internal, stable, and global causes. The extent to which people tend to be optimistic varies across individuals (e.g., Carver and Scheier, 2002; Icekson and Pines, 2012). Additionally, situational factors influence optimism. For example, asking people to generate positive thoughts about their future boosts optimism temporarily (Fosnaugh et al., 2009).

Optimistic individuals believe they can overcome obstacles and perceive difficult tasks as challenges rather than threats (Smith et al., 1993; Chang, 1998). Optimism stimulates persistence in goal pursuit (Brown and Marshall, 2001), and enhances psychological and physical adjustment to stressful events (Carver et al., 2010). Beyond the obvious benefits to health and wellbeing, optimism enhances performance in the academic (Chemers et al., 2001; Nes et al., 2009), athletic (Gould et al., 2002; Gordon, 2008), work (Seligman and Schulman, 1986; Kluemper et al., 2009), and creative domain (Rego et al., 2012).

Despite the notable advantages of having a positive lookout, overly positive expectations sometimes have negative consequences and lead to poorer performance. Optimistic individuals tend to underestimate potential threats and obstacles, take risks, and persist in investing in hopeless endeavors (Felton et al., 2003; Trevelyan, 2008; Hmieleski and Baron, 2009). Optimists, for example, are more likely than pessimists to continue gambling after losing money (Gibson and Sanbonmatsu, 2004).

Optimism thus can have positive but also negative effects on performance. Here, we propose that for creative performance the positive effects of optimism are particularly likely to manifest themselves when people are avoidance motivated. When people are approach motivated, these positive effects should be less prominent, and negative effects of over-optimism are more likely to occur. It is likely that there is a negative relation between trait avoidance motivation and trait optimism. However, even when people are avoidance motivated (due to individual differences or situational cues), levels of optimism about successfully avoiding specific negative outcomes vary across situations. For example, someone may be rather optimistic about the likelihood of not failing an exam, but less optimistic about the likelihood of not getting hurt on a skiing trip. In the following, we review the psychological processes that are evoked by avoidance motivation, and discuss how optimism may reduce negative effects of these processes on creativity.

## Optimism, a recipe for creativity under avoidance motivation

Compared to approach motivation, avoidance motivation is associated with a host of cognitive, affective, and volitional processes that can undermine creative performance (for a thorough review of these processes see Elliot et al., 2013). Here we discuss why these processes influence creativity, and how optimism impacts each of these processes and may thereby mitigate negative effects of avoidance motivation.

### Cognitive processes

According to* Cognitive Appraisal* theories (Lazarus and Folkman, 1984) demanding tasks or situations are evaluated according to subjective perceptions of demands and available resources. When demands exceed resources, situations are evaluated as threats. However, when situations are taxing yet rewarding, they are perceived as challenges. When people are approach motivated, they tend to appraise situations in terms of challenges, whereas when they are avoidance motivated they tend to appraise situations in terms of threats. Approach motivation and the associated challenge appraisals evoke flexible and associative information processing, which enhances creativity (Baas et al., 2008; Gutnick et al., 2012). Avoidance motivation and the associated threat appraisals, on the other hand, evoke persistent and systematic information processing (Friedman and Förster, 2002; Friedman and Elliot, 2008). This persistent processing style does not render creativity impossible, but makes it more difficult and effortful. In order to achieve creative output, people need to exert focused effort to compensate for their inflexible processing style (Roskes et al., 2012, 2013). Therefore, avoidance motivation often reduces creativity (Friedman and Förster, 2002, 2005; Mehta and Zhu, 2009). In the best case scenario, when avoidance motivated people are willing to go the extra mile and invest effort into creative performance, they are as creative as approach motivated people (at least in the short term) but end up tired and depleted (Roskes et al., 2012; Ståhl et al., 2012). Threat appraisals, thus, are suboptimal when striving for creative output.

Optimism increases the likelihood of perceiving demanding situations as challenging rather than threatening (Smith et al., 1993; Chang, 1998). Consequently, when people are avoidance motivated, optimism about the likelihood of avoiding negative outcomes may reduce threat appraisals and enhance challenge appraisals (e.g., using cognitive therapy; Gardner et al., 2005). These reduced threat and enhanced challenge appraisals, in turn, should stimulate cognitive flexibility, thereby increasing creativity (Gutnick et al., 2012). When people are approach motivated, they already tend to appraise situations as challenges and engage in flexible processing. Therefore, optimistic beliefs about their abilities to attain positive outcomes should not enhance their creativity as much.

### Affective processes

Approach motivation is experienced as a positive state in which positive emotions such as joy and excitement are easily elicited (Pekrun et al., 2009).

In contrast, striving to avert negative outcomes evokes anxiety, worry, and fear of failure (Gable et al., 2000; Eysenck et al., 2007). These negative emotions narrow people’s attention scope and impede cognitive flexibility (Baas et al., 2008; De Dreu et al., 2008). Optimism may enhance creativity among avoidance motivated people by moderating the hedonic tone of affective reactions, thereby broadening the attention scope. Optimism is inversely related to tension and worry. First, it influences neuroendocrine regulation by decreasing the secretion of stress hormones (Lai et al., 2005; Endrighi et al., 2011). For example, optimism decreases the association between stress perceptions and elevated levels of cortisol (Jobin et al., 2013). Second, optimism intensifies positive emotions such as enthusiasm and happiness (Hodges and Winstanley, 2012) and attenuates negative emotions such as sadness and fear (Lucas et al., 1996; Siddique et al., 2006). When people are avoidance motivated, stimulating optimism about the likelihood of achieving avoidance goals can mitigate negative affect and in doing so increase creativity. Again, when people are approach motivated, and are already experiencing little negative affect, this positive effect of optimism should be reduced.

### Volitional processes

When people strive to avoid negative outcomes (e.g., avoid losing one’s job, embarrassing oneself, or performing worse than others), there is no positive end state to look forward to. The best outcome of avoidance goal achievement is the absence of negative outcomes, which can be important, but doesn’t provide much fuel for excitement or intrinsic motivation. Avoidance goal striving can therefore be experienced as an obligation—something one *has* to do (Higgins, 1997; Carver et al., 2000; Ryan and Deci, 2006). For creativity, intrinsic motivation, the feeling that one’s actions have meaning and purpose, is crucial (Amabile, 1983; Friedman, 2009). The low intrinsic motivation involved in avoidance goal pursuit, is thus another factor undermining creativity. Additionally, because creativity is relatively effortful for them, avoidance motivated people only invest in creativity when this is perceived as necessary for avoiding failure or averting losses (Roskes et al., 2012). When people are avoidance motivated, they need to be actively stimulated and convinced that their creative efforts will be useful. Finally, when people focus on avoiding negative outcomes rather than achieving positive ones, they are more liable to engage in simple tasks in which failure is unlikely and to withdraw effort (i.e., “self-handicapping”) to protect themselves from demonstrating low ability (Alicke and Sedikides, 2009; Righetti et al., 2011).

Optimism may buffer against the negative effects of avoidance motivation on these volitional processes. Optimism enhances proactive and persistent goal pursuit, and reduces the urge to disengage or give up. For example, highly optimistic HIV, cancer, and cardiac patients were more likely to seek information about their condition and make plans for recovery than less optimistic patients (Carver et al., 2010; Forgeard and Seligman, 2012). Moreover, optimism increases willingness to invest effort and persist, even when facing adversity (Carver et al., 2010). Optimism may thus increase engagement, proactive goal pursuit, and willingness to invest effort, which should particularly benefit creativity when people are avoidance motivated.

To summarize, avoidance motivation is associated with cognitive, affective, and volitional processes that can be problematic when striving for creativity. Due to its impact on each of these processes, optimism is a good candidate for stimulating creativity under avoidance motivation.

## Optimism, a suppressor of creativity under approach motivation?

Both approach motivation and optimism are associated with challenge appraisals, cognitive flexibility, excitement, and intrinsic motivation, which are conducive to high levels of creativity. Intuitively, we may therefore expect the combination of approach motivation and optimism to be optimal for creativity. However, a closer look at the processes involved suggests that this may not always be the case. When people are approach motivated, they tend to focus on potential gains and overlook obstacles and dangers. They pay less attention to threatening cues and feel more confident about achieving their goals (Elliot, 1999). Under avoidance motivation, optimism may provide balance, and help to see beyond threats and difficulties. However, under approach motivation optimism may tip the scales into the direction of over-optimism with its associated negative consequences.

Indeed, high levels of optimism are related to an attentional bias toward positive stimuli, ignoring contradictions, and neglecting threatening information (Segerstrom, 2001; Geers and Lassiter, 2002; Geers et al., 2003; Isaacowitz, 2005). This prevents people from taking precautionary behaviors. For example, Weinstein and Lyon (1999) found that highly optimistic homeowners living in a high radon risk area underestimated their personal health risks, and consequently were less likely to purchase radon test kits. Moreover, over-optimism can be detrimental for performance; for instance, overly optimistic students perform worse than moderately optimistic students. Similarly, negative effects of high levels of optimism on academic performance only occurred for students that have low conscientiousness, a personality trait characterized by self-discipline (Icekson and Kaplan, working paper). Additionally, discounting of negative feedback when working on creative tasks, may lead to the generation of original, but not very useful ideas. This is problematic, because to be considered creative, ideas need to be both novel and useful. These pieces of evidence suggest that the risk for over-optimism may be greater when people are approach rather than avoidance motivated.

When people are approach motivated, high optimism may undermine creative performance, due to reduced preparation, effort, and discounting of negative but relevant information. In sum, we suggest that the risk for unrealistic positive expectations may be larger when people are approach motivated and have their eyes on the prize, rather than on the obstacles along the way. Consequently, their creativity may be undermined.

## Implications and directions for future research

Avoidance motivation makes creativity difficult and often undermines it, due to a variety of psychological processes that are discussed in this paper. Thorough understanding of the processes that enhance and impair creativity under approach and avoidance motivation, will enhance creativity and motivation theory, and enable to develop interventions aimed at stimulating creativity (also see Roskes, in press; Roskes et al., in press).

From a practical perspective, eliciting optimism among people who are avoidance motivated should stimulate creativity. This can be done by designing environments in ways that enhance optimism, or by directly training individuals to adopt more optimistic views. For example, managers can adapt their leadership style to communicate their positive vision to followers. Such positive expectations of leaders can instill optimism in their subordinates (McColl-Kennedy and Anderson, 2002). Optimism can also be stimulated by individual level interventions, as demonstrated by cognitive therapy or by exercises like “the best possible self” (BPS), which requires to envision oneself in an imaginary future where everything turned out in the most optimal way (King, 2001; Gardner et al., 2005; Meevissen et al., 2011). When people are approach motivated, stimulating optimism is less needed to stimulate creativity. First, because approach motivated people are inherently more likely to be optimistic, and second because they may be at risk for the negative consequences of over-optimism. While optimism should be boosted among avoidance motivated people, approach motivated people may instead benefit from being cautioned.

From a theoretical perspective, it is important to study the processes that play a role in predicting creativity under approach and avoidance motivation more thoroughly. For clarity, cognitive, affective, and volitional processes involved in creativity have been discussed separately in this paper. However, many of these processes are inherently interdependent. Threat appraisals, for example, are closely related to negative emotions (Lazarus, 1999), physiological stress responses (Seery, 2011), and reduced intrinsic motivation (Drach-Zahavy and Erez, 2002), whereas challenge appraisals are related to positive affect (Skinner and Brewer, 2002) and enhanced effort and motivation (Drach-Zahavy and Erez, 2002). Future research is needed to disentangle the roles of the various processes in shaping effects of approach and avoidance motivation on creativity, and to deepen our understanding regarding optimal levels of optimism. This will help to build theory, and to identify potential factors that impact these processes and can ameliorate the negative effects of avoidance motivation on creativity.

## Conclusion

Focusing on avoiding failure or negative outcomes can undermine creativity, due to cognitive (e.g., threat appraisals), affective (e.g., anxiety), and volitional processes (e.g., low intrinsic motivation). This can be problematic for people who are avoidance motivated by nature and in situations in which threats or potential losses are salient. Optimism positively impacts each of the problematic processes evoked by avoidance motivation, and should thus reduce the undermining effect on creativity. We further suggest that optimism may suppress the positive relation between approach motivation and creativity. More broadly, we propose that for effectively stimulating creative performance, it is important to address the core psychological processes underlying creativity and identify factors that influence these processes.

## Conflict of interest statement

The authors declare that the research was conducted in the absence of any commercial or financial relationships that could be construed as a potential conflict of interest.
